# A Novel Sugar Transporter from *Dianthus spiculifolius*, *DsSWEET12*, Affects Sugar Metabolism and Confers Osmotic and Oxidative Stress Tolerance in *Arabidopsis*

**DOI:** 10.3390/ijms19020497

**Published:** 2018-02-07

**Authors:** Aimin Zhou, Hongping Ma, Shuang Feng, Shufang Gong, Jingang Wang

**Affiliations:** 1 College of Horticulture and Landscape Architecture, Northeast Agricultural University, Harbin 150030, China; aiminzhou@neau.edu.cn (A.Z.); HongpingMa@aliyun.com (H.M.); shufanggong@neau.edu.cn (S.G.); 2 Key Laboratory of Saline-Alkali Vegetation Ecology Restoration in Oil Field (SAVER), Ministry of Education, Alkali Soil Natural Environmental Science Center (ASNESC), Northeast Forestry University, Harbin 150040, China; shuangfeng1986@aliyun.com

**Keywords:** sugar transporter, *DsSWEET12*, *Dianthus spiculifolius*, sugar metabolism, osmotic and oxidative stress

## Abstract

Plant SWEETs (sugars will eventually be exported transporters) play a role in plant growth and plant response to biotic and abiotic stresses. In the present study, *DsSWEET12* from *Dianthus spiculifolius* was identified and characterized. Real-time quantitative PCR analysis revealed that *DsSWEET12* expression was induced by sucrose starvation, mannitol, and hydrogen peroxide. Colocalization experiment showed that the DsSWEET12-GFP fusion protein was localized to the plasma membrane, which was labeled with FM4-64 dye, in *Arabidopsis* and suspension cells of *D. spiculifolius*. Compared to wild type plants, transgenic *Arabidopsis* seedlings overexpressing *DsSWEET12* have longer roots and have a greater fresh weight, which depends on sucrose content. Furthermore, a relative root length analysis showed that transgenic *Arabidopsis* showed higher tolerance to osmotic and oxidative stresses. Finally, a sugar content analysis showed that the sucrose content in transgenic *Arabidopsis* was less than that in the wild type, while fructose and glucose contents were higher than those in the wild type. Taken together, our results suggest that *DsSWEET12* plays an important role in seedling growth and plant response to osmotic and oxidative stress in *Arabidopsis* by influencing sugar metabolism.

## 1. Introduction

Plants produce sugar in leaf mesophyll cells by photosynthesis. These sugars, mostly sucrose, are imported via bundle sheaths into phloem cells and transported to growing parts of the plant [1]. Sugar, mainly sucrose, fructose, and glucose, is a source of both carbon and energy, and its transport, distribution, and utilization play key roles in the regulation of plant growth and development and plant responses to biotic and abiotic stresses [2]. Sugar translocation between cell compartments, cells, and organs depends on sugar transporters [3]. Plant SWEETs (sugars will eventually be exported transporters) are a class of H^+^-independent mono- and disaccharide transporters [1,4]. They comprise a large gene family and have been identified from several species including *Arabidopsis thaliana* [5], *Oryza sativa* [6], *Sorghum bicolor* [7], and *Citrus sinensis* [8].

In *Arabidopsis*, the AtSWEET sugar transporter family contains 17 members. AtSWEET1 was the first plant SWEET to be characterized; it acts as a glucose uniporter in endoplasmic reticulum and plasma membranes [9]. AtSWEET4 is localized on the plasma membrane of axial tissue and plays an important role in the accumulation of glucose and fructose [10]. AtSWEET9, a nectary-specific sugar transporter, is necessary for nectar secretion [11]. AtSWEET16 and AtSWEET17 are fructose transporters localized on the vacuolar membrane and participate in the regulation of fructose levels [12,13,14,15]. AtSWEET11 and AtSWEET12 have been identified as sugar transporters and are localized to the plasma membrane of phloem parenchyma cells and play a key role in sucrose phloem loading [1,16,17]. Furthermore, AtSWEET11, AtSWEET12, and AtSWEET15 have a distinct roles in seed filling [18]. The rice ortholog of AtSWEET11 and AtSWEET12, OsSWEET12, causes pathogen susceptibility possibly by sugar leakage from infected cells [19]. Heterologous expression in *Xenopus laevis* oocytes showed that, in addition to transporting sucrose, AtSWEET11 and AtSWEET12 have the ability to transport fructose and glucose [16]. The remaining family members have yet to be characterized. These findings indicate the importance and complexity of SWEET functions. However, few SWEETs have been identified and characterized in non-model wild plants.

*Dianthus spiculifolius* Schur, a perennial herbaceous plant and a member of the Caryophyllaceae, shows strong resistance to cold and drought stress [20]. In this study, we identified the *DsSWEET12* gene from transcriptome data of mannitol-treated *D. spiculifolius*. *DsSWEET12* expression was investigated by real time quantitative PCR (qPCR). Subcellular localization of DsSWEET12 in plant cells was investigated by using green fluorescent protein (GFP) as a marker combined with FM4-64 staining. Finally, transgenic *Arabidopsis* was used to investigate the function of DsSWEET12 in plant growth and stress response.

## 2. Results

### 2.1. Sequence Analysis of DsSWEET12

**Figure 1:**
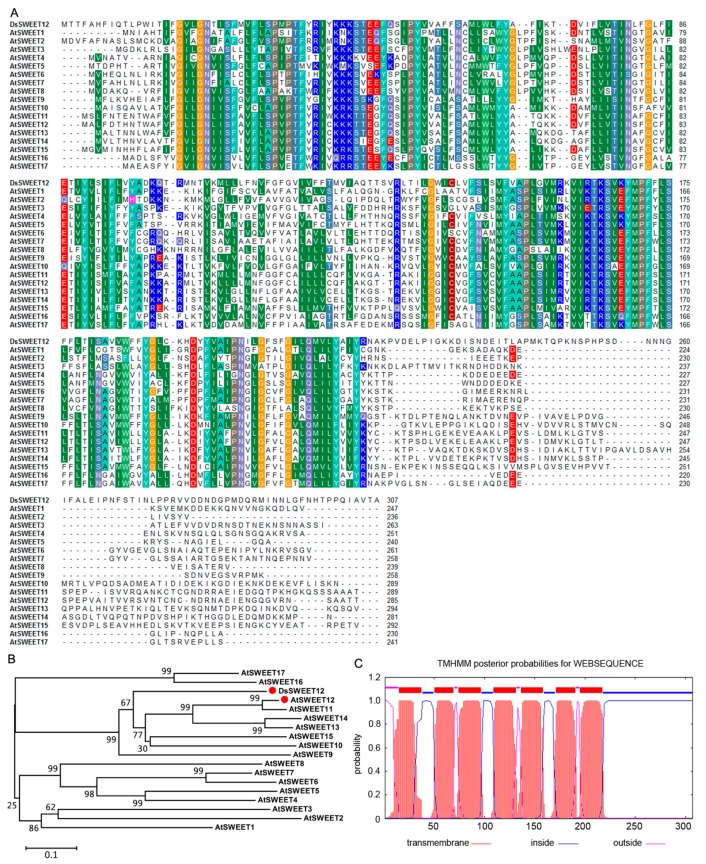
Sequence analysis of DsSWEET12. Amino acid sequence alignment (**A**) and phylogenetic tree (**B**) of DsSWEET12 with AtSWEET family (AtSWEET1 to AtSWEET17) from *Arabidopsis*. The same color backgrounds indicate identical or highly similar residues in each sequence. (**C**) Putative transmembrane domains of DsSWEET12.

The full-length *DsSWEET12* cDNA sequence was observed to contain a 924-bp ORF, which was predicted to encode a protein of 307 amino acids and a molecular mass of 35.08 kDa. The amino acid sequence of DsSWEET12 was determined to be similar (41.96% identity) to that of the AtSWEET family (AtSWEET1 to AtSWEET17) (Figure 1A). The phylogenetic tree constructed using the SWEET sequences revealed that DsSWEET12 is most closely related to AtSWEET12 (Figure 1B). DsSWEET12 was predicted using the TMHMM algorithm to have seven transmembrane regions (Figure 1C).

### 2.2. Expression and Subcellular Localization of DsSWEET12

**Figure 2:**
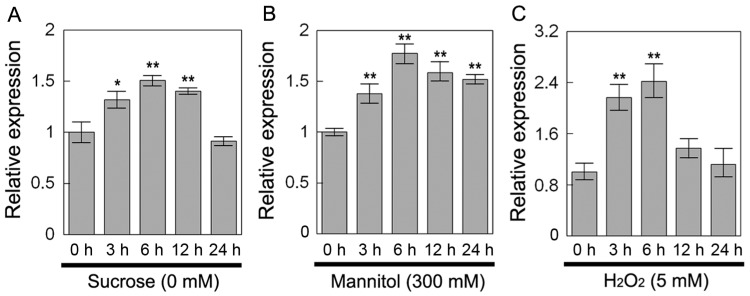
Expression of *DsSWEET12*. Expression analysis of *DsSWEET12* under sucrose starvation and other various stresses. One-week-old *D. spiculifolius* seedlings were treated with (**A**) sucrose starvation (0 mM), (**B**) mannitol (300 mM), (**C**) and H_2_O_2_ (5 mM) for 0, 3, 6, 12, and 24 h. *DsActin* was used as an internal control, and the transcript level in the untreated seedlings was set as 1.0. Asterisks indicate significant difference between untreated and stress-treated seedlings (* *p* < 0.05; ** *p* < 0.01; Student’s *t* test). Error bars show the SD of the values from three replicates.

*DsSWEET12* expression was first investigated under sucrose starvation and under osmotic and oxidative stress using qPCR. Under sucrose (0 mM) starvation, *DsSWEET12* expression was induced within 3 to 12 h of treatment, peaked at 6 h, and then decreased to its original level at 24 h (Figure 2A). Under mannitol (300 mM) treatment, *DsSWEET12* expression was induced within 3 to 24 h of treatment, and peaked at 6 h (Figure 2B). Under H_2_O_2_ (5 mM) treatment, *DsSWEET12* expression peaked at 6 h, and then declined at 12 and 24 h after treatment (Figure 2C). This result suggests that the expression of *DsSWEET12* was affected by sucrose starvation, mannitol stress, and H_2_O_2_ stress at different time points.

**Figure 3:**
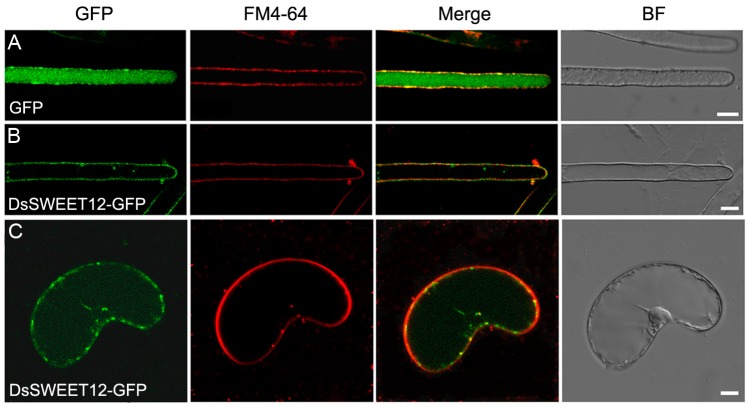
Colocalization of DsSWEET12-GFP with FM4-64 in *Arabidopsis* root hair cells and *D. spiculifolius* suspension cells. *Arabidopsis* root hairs stably expressing green fluorescent protein (GFP) (**A**) or DsSWEET12-GFP (**B**) and *D. spiculifolius* suspension cells transiently expressing DsSWEET12-GFP (**C**) were incubated for 1 min with 4 μM FM4-64. GFP fluorescence is green, and FM4-64 is red. Merge is created by merging the GFP and FM4-64 fluorescence images. BF are bright field images. Scale bars = 10 µm.

Further, localization of DsSWEET12 in plant cells was examined using GFP as a fusion protein marker combined with FM4-64 dye. Confocal images showed that GFP alone was localized in the cytoplasm of root hair cells of *Arabidopsis* stably expressing GFP (Figure 3A). In *Arabidopsis*, root hair cells stably expressing DsSWEET12-GFP, the GFP signals were observed in the cell periphery, which were colocalized with the plasma membrane marker dye FM4-64 (staining for 1 min) (Figure 3B). In *D. spiculifolius* suspension cells transiently expressing DsSWEET12-GFP, the GFP signal was also colocalized with FM4-64 to the cell periphery (Figure 3C). In addition, a small number of punctate GFP signals were observed in cells (Figure 3B,C). This result suggests that DsSWEET12-GFP was mainly localized at the plasma membrane in plant cells.

### 2.3. Overexpression of DsSWEET12 in Arabidopsis Affects Seedling Growth and Sugar Metabolism

**Figure 4:**
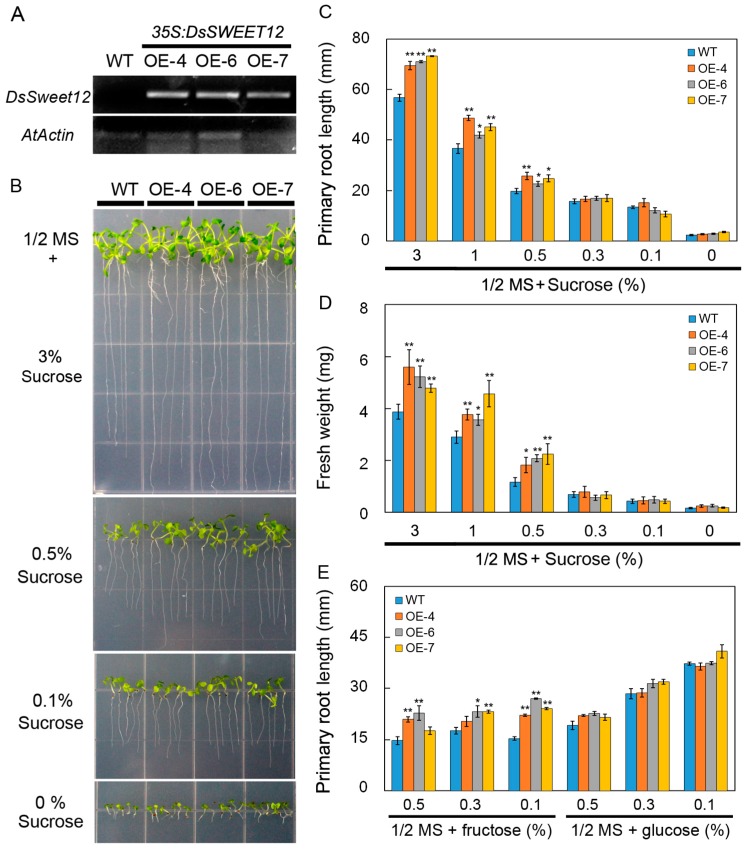
Growth comparison of wild type (WT) and *DsSWEET12* transgenic *Arabidopsis* on 1/2 MS medium containing different concentrations of sugar. (**A**) Semi-quantitative PCR analysis of *DsSWEET12* expression in WT and transgenic *Arabidopsis* lines (OE-4, OE-6, and OE-7). (**B**–**D**) Seedling growth (**B**), root length (**C**), and fresh weight (**D**) of WT and three transgenic lines on 1/2 MS medium (3% sucrose) containing different concentrations of sucrose. (**E**) Root length of WT and three transgenic lines on 1/2 MS (sucrose free) medium supplemented with different concentrations of fructose or glucose. Asterisks indicate significant difference between WT and transgenic lines (* *p* < 0.05; ** *p* < 0.01; Student’s *t* test). Error bars show the SE of the values from three replicates.

**Figure 5:**
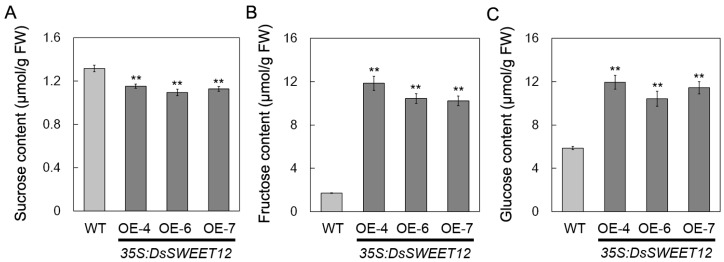
Sucrose (**A**), fructose (**B**), and glucose (**C**) content in wild type (WT) and *DsSWEET12* transgenic *Arabidopsis* (OE-4, OE-6, and OE-7) seedlings. The sugar content was measured from seedlings grown on 1/2 MS (Murashige and Skoog; 3% sucrose) medium for 3 weeks. Asterisks indicate significant differences between WT and transgenic lines (** *p* < 0.01; Student’s *t* test). Error bars show SE of the values from three replicates.

Transgenic *Arabidopsis* plants overexpressing *DsSWEET12* driven by a CaMV35S promoter were generated to evaluate the role of *DsSWEET12* in plant growth. The expression of *DsSWEET12* in T3 transgenic lines was confirmed by semi-quantitative reverse transcription PCR (Figure 4A). In the 1/2 MS medium (3% sucrose), root length and fresh weight of transgenic seedlings were significantly greater than those of the wild type (WT). On the basis of 1/2 MS salt, differences in root length and fresh weight between transgenic and WT seedlings gradually decreased and even disappeared as the sucrose concentration decreased (Figure 4B–D). In addition, on 1/2 MS medium (sucrose free) supplemented with fructose (not glucose), the roots of the transgenic seedlings grew faster than those of WT plants (Figure 4E). Further, the sugar content of the transgenic and WT seedlings was compared. Compared with the WT, the sucrose content in the transgenic seedlings was significantly reduced (Figure 5A), while the contents of fructose and glucose increased significantly (Figure 5B,C). These results suggest that overexpression of *DsSWEET12* in *Arabidopsis* affects seedling growth and sugar metabolism.

### 2.4. Overexpression of DsSWEET12 in Arabidopsis Improves Tolerance to Osmotic and Oxidative Stresses

**Figure 6:**
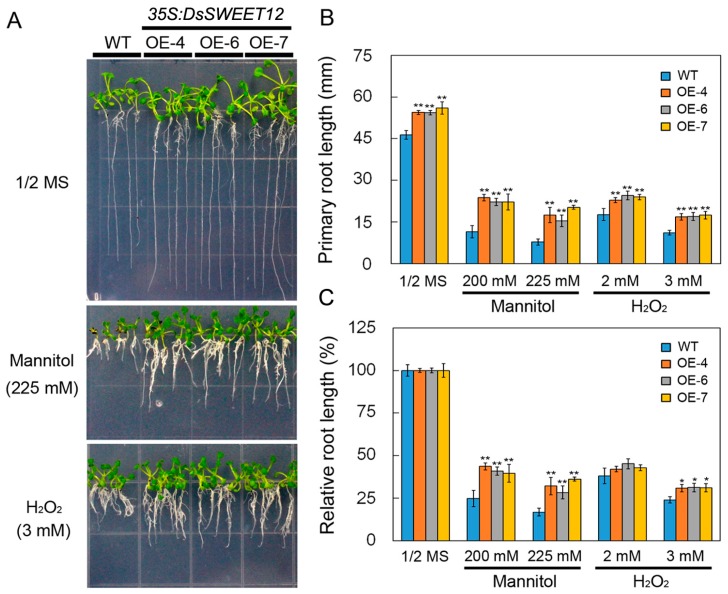
Phenotypes of wild type (WT) and *DsSWEET12* transgenic *Arabidopsis* under various stresses. Seedling growth (**A**), root length (**B**), and relative root length (**C**) of WT and three transgenic lines on 1/2 MS (Murashige and Skoog) medium supplemented with mannitol (200 and 225 mM), H_2_O_2_ (2 and 3 mM), and NaCl (125 mM). Asterisks indicate significant differences between WT and transgenic lines (* *p* < 0.05; ** *p* < 0.01; Student’s *t* test). Error bars show the SE of the values from three replicates.

Phenotypes of transgenic and WT seedlings under osmotic and oxidative stress were compared. On 1/2 MS or 1/2 MS supplemented with mannitol (200 and 225 mM) and H_2_O_2_ (2 and 3 mM), the root length of transgenic seedlings was significantly greater than that of WT plants (Figure 6A,B). However, relative root length analysis showed that root growth of transgenic seedlings was significantly less inhibited by mannitol (200 and 225 mM) and H_2_O_2_ (3 mM) stress compared with the WT (Figure 6C). This result suggests that overexpression of *DsSWEET12* in *Arabidopsis* not only promotes seedling root growth, but also improves tolerance to osmotic and oxidative stresses.

## 3. Discussion

In *Arabidopsis*, AtSWEET12 is localized to the plasma membrane of phloem parenchyma cells and mediates phloem loading of sucrose [17]. In our study, qPCR analysis showed that *DsSWEET12* expression was induced by sucrose starvation (Figure 2A). Colocalization experiments confirmed that DsSWEET12-GFP was mainly localized to the plasma membrane, which was labeled with FM4-64 dye, in *Arabidopsis* root hair and *D. spiculifolius* suspension cells (Figure 3B,C). In addition, intracellular punctate GFP signals were also observed (Figure 3B,C), suggesting that DsSWEET12-GFP may also be localized to other subcellular organelles in addition to plasma membrane. Phenotypic analysis showed that overexpression of *DsSWEET12* in *Arabidopsis* promoted seedling growth, which depended on sucrose content (Figure 4A–D). Furthermore, sugar content analysis showed that the sucrose content in transgenic seedlings was lower than that in WT seedlings, while fructose and glucose contents were higher (Figure 5), indicating that overexpression of *DsSWEET12* affected sugar metabolism in *Arabidopsis*. We speculated that the change in sugar content may be caused by the expression of 35S-driven *DsSWEET12* in the whole plant. Overexpression of *DsSWEET12* may affect sugar transport and indirectly affect sugar metabolism. Sugar, a source of both carbon and energy, is necessary for plant cell growth [3]. Taken together, our results suggest that DsSWEET12 is a sugar transporter in the plasma membrane, and its overexpression affects seedling growth and sugar metabolism in *Arabidopsis*. In the absence of sucrose, the addition of external fructose also affected the root growth of transgenic seedlings (Figure 4E), indicating that DsSWEET12 may play a role in the transport or utilization of fructose in addition to sucrose transport. Previous reports have shown that AtSWEET12 also has the ability to transport fructose [16].

qPCR analysis showed that the expression of *DsSWEET12* was also induced by mannitol and H_2_O_2_ treatment (Figure 2B,C). Relative root length analysis showed that mannitol (200 and 225 mM) and H_2_O_2_ (3 mM) inhibited root growth in transgenic seedlings significantly less than in WT seedlings (Figure 6C), indicating that overexpression of *DsSWEET12* not only affected seedling growth, but also improved *Arabidopsis* tolerance for osmotic and oxidative stress. The results of the sugar content analysis showed that the total sugar content (the reduction in sucrose was less than the increase in fructose and glucose) in the transgenic seedlings was higher than that in the WT seedlings (Figure 5). In addition to being a carbon source, sugar also acts as an osmotic regulator and can affect the osmotic balance of plant cells [2]. H_2_O_2_, which is a reactive oxygen species (ROS), can lead to oxidative stress within plant cells. There have been studies showing that sugar signaling and sugar-modulated gene expression are related to the control of oxidative stress by participating in the ROS scavenging pathway [21]. Thus, we speculate that overexpression of *DsSWEET12* confers tolerance to osmotic and oxidative stress by affecting sugar metabolism in *Arabidopsis*.

## 4. Materials and Methods

### 4.1. Identification of DsSWEET12 and Sequence Analysis

Based on sequence similarity, the *DsSWEET12* gene (GenBank accession number: MG737823) was identified from transcriptome sequencing data of mannitol-treated *D. spiculifolius*. Amino acid sequences of DsSWEET12 and its homologs were aligned using ClustalW, and transmembrane domains in DsSWEET12 were predicted by the TMHMM algorithm (available online: http://www.cbs.dtu.dk/services/TMHMM/). The phylogenetic tree was constructed by the neighbor-joining method using molecular evolutionary genetics analysis (MEGA) 4.1 software (available online: http://www.megasoftware.net/) with 1000 bootstrap replicates, the bootstrap scores <50% were deleted.

### 4.2. Plant Material and Growth Conditions

*Dianthus spiculifolius* and *A. thaliana* seeds were surface sterilized and stratified at 4 °C for 2 days in the dark. After germination, the seedlings were grown on 1/2 strength Murashige and Skoog (MS) medium (3% sucrose, 1% agar: pH 5.8) under a 12 h light/12 h dark photoperiod (100 μmol m^−2^ s^−1^ light intensity) at 22 °C. All *A. thaliana* plants used in this study belonged to the Columbia-0 (Col-0) ecotype.

For abiotic stress treatments, 1-week-old *D. spiculifolius* seedlings were exposed to 1/2 MS (3% sucrose) supplemented with 300 mM mannitol, and 5 mM H_2_O_2_ treatments or 1/2 MS (sucrose free). At least 10 seedlings from each treatment were harvested and pooled at different time points (0, 3, 6, 12, or 24 h after treatment), frozen immediately in liquid nitrogen, and stored at −80 °C for RNA preparation.

### 4.3. RNA Extraction and Quantitative Real-Time PCR (qPCR) Analyses

Total RNA was extracted using an RNeasy^®^ Mini Kit (Qiagen, Valencia, CA, USA), according to the manufacturer’s instructions. First-strand cDNA was synthesized from 1 µg of total RNA with the M-MLV RTase cDNA Synthesis Kit (TaKaRa, Shiga, Japan). Real-time quantitative PCR (qPCR) analysis was performed using SYBR^®^ Green Mix (Agilent Technologies, Palo Alto, CA, USA) in an optical 96-well plate on an Mx3000P system (Agilent Technologies). Three biological replicates and three technical replicates were performed for each analysis. The primers used in this study are shown in Table 1.

### 4.4. Vector Construction and Plant Transformation

For the construction of the pBI121-DsSWEET12 construct, the open reading frame (ORF) of *DsSWEET12* was amplified by PCR and cloned at the *BamH*I and *Sac*I sites of the pBI121 vector. To construct the GFP fusion genes, the ORF of *DsSWEET12*, without the stop codon, was amplified by PCR and cloned at the *BamH*I and *Kpn*I sites of the pBI121-GFP vector. These pBI121-GFP constructs have been described elsewhere [22]. The accuracy of the above constructs was confirmed by sequencing and the specific primers used in this study are shown in Table 1.

The constructs were transformed into *Agrobacterium tumefaciens* strain EHA105 for plant transformation; *Arabidopsis* was transformed using the floral dip method [23]. The transgenic plants were selected on 1/2 MS medium containing 30 μg mL^−1^ kanamycin. Expression of *DsSWEET12* in the transgenic lines was assessed by semi-quantitative reverse transcription PCR analyses. The T3 generation was used for the analyses.

For suspension cell transfection, *D. spiculifolius* seeds were grown in modified MS medium (3% sucrose, 1.0 mg mL^−1^ 6-benzyl aminopurine, 2.5 mg mL^−1^ 2,4-dichlorophenoxyacetic acid, 1% agar; pH 5.8) and incubated for 14 days at 22 °C to induce calli. *A. tumefaciens*-mediated transfection of suspension cells was performed according to a previously described procedure [22].

### 4.5. Confocal Laser Scanning Microscopy

Four-day-old seedlings grown on vertical 1/2 MS agar plates and *D. spiculifolius* suspension cells were incubated in 1 mL of liquid 1/2 MS medium (0.5% sucrose, pH 5.8) that contained 4 μM FM4-64 (Invitrogen, Carlsbad, CA, USA) for 1 min at room temperature. The roots of transgenic *Arabidopsis* seedlings were washed twice with liquid 1/2 MS medium immediately before confocal laser scanning microscopy (CLSM; Nikon, A1, Tokyo, Japan). GFP signals were detected using a 500–530 nm emission filter. FM4-64 signals were detected using a 620–680 nm emission filter.

### 4.6. Sugar Starvation and Stress Tolerance Assay

For sugar starvation and stress tolerance tests, *Arabidopsis* seeds were treated at 4 °C for 2 days and then grown for 10 or 14 days on 1/2 MS medium (sucrose free) containing different concentrations of sucrose (3%, 1%, 0.5%, 0.3%, 0.1%, and 0%), fructose (0.5%, 0.3%, and 0.1%), and glucose (0.5%, 0.3%, and 0.1%) or 1/2 MS medium (3% sucrose) supplemented with mannitol (200 or 225 mM), and H_2_O_2_ (2 or 3 mM) before measurements of root length and fresh weight were taken. The experiment was replicated three times.

### 4.7. Sugar Content Analysis

To measure sugars, *Arabidopsis* seedlings (0.2 g fresh weight, FW) were homogenized in 2 mL of hyperpure water, centrifuged at 8000× *g* for 10 min at 4 °C. After passing through a 0.22 mm filter, a 10 μL sample was injected into a Kro-masil^®^ NH2 column (4.6 × 250 mm), and sugar contents were analyzed by high performance liquid chromatography (HPLC) (Waters 510, Waters Associates Inc., Milford, MA, USA).

### 4.8. Statistical Analyses

All experiments were done with three independent biological and three technical replicates. Data were analyzed with a one-way analysis of variance by SPSS, and statistically significant differences were calculated with a Student’s *t*-test, with *p* < 0.05 (*) and *p* < 0.01 (**) used as the thresholds for significance.

## Figures and Tables

**Table 1:** List of primers used in this study.

Primer Name	Primer Sequence (5′→3′)	Purpose
DsSWEET12-qF	CAAAGCATTCCCTATGTGGTG	qPCR
DsSWEET12-qR	TAGTTTGCTTGTCGGCGTAGA	qPCR
DsActin-qF	CGGTGGCTCTATCCTCGCTT	qPCR
DsActin-qR	TTCCTGTGGACGATTGACGG	qPCR
AtActin1-F (AT2G37620)	GAAAATGGCTGATGGTGAAG	RT-PCR
AtActin1-R	CTCATAGATAGGAACAGTGTGGC	RT-PCR
DsSWEET12 (BamHI)-F	GGATCCATGACTACTTTTGCTCACTTC	Cloning and Subcellular localization
DsSWEET12 (SacI)-R	GAGCTCTTAGGCAGTCACGGCAATT	Cloning
DsSWEET12 (KpnI)-R	GGTACCGCAGTCACGGCAATTTGAG	Subcellular localization
